# Mental health service use among Filipino American and Korean American young adults during the COVID‐19 pandemic

**DOI:** 10.1002/ajcp.70043

**Published:** 2025-12-26

**Authors:** Michael Park, Eunseok Jeong, Nari Yoo, Yoonsun Choi, Leopoldo J. Cabassa, Miwa Yasui, David Takeuchi

**Affiliations:** ^1^ School of Social Work, Rutgers The State University of New Jersey New Brunswick New Jersey USA; ^2^ Crown Family School of Social Work, Policy and Practice University of Chicago Chicago Illinois USA; ^3^ School of Social Work University of Michigan Ann Arbor Michigan USA; ^4^ Brown School and the Center for Mental Health Services Research Washington University in St. Louis St. Louis Missouri USA; ^5^ School of Social Work University of Washington Seattle Washington USA

**Keywords:** COVID‐19, Filipino Americans, Korean Americans, mental health service use, young adults

## Abstract

Despite the heightened mental health challenges amid rising Anti‐Asian sentiment, Asian Americans have significantly underutilized mental health services, a trend that persisted even before the COVID‐19 pandemic. Although considerable efforts have been made to understand how various factors are related to mental health service use in this population, research integrating these multiple factors in a single study, with a specific focus on ethnic disaggregation, remains limited. Using a cross‐sectional Study of Filipino and Korean American young adults (*M*_age_ = 21.37, US‐born = 65.03%), we examined the combined impact of individual, familial, and ethnic‐cultural, immigrant, and racial stereotype factors on their mental health service utilization through hierarchical logistic regressions. Depressive symptoms, being female, and less stigma associated with mental health care were significantly associated with more service use regardless of ethnicity. Notably, primarily speaking English or both English and ethnic language equally at home (as opposed to an ethnic language) was significantly associated with more service use among US‐born Filipino Americans. Conversely, the internalized model minority stereotype was significantly associated with less service use among Korean Americans. This study underscores the importance of developing effective mental health interventions tailored to both shared and unique determinants within diverse Asian American populations.

## Highlights

- This study examines patterns of mental health service use among Filipino American and Korean American young adults during the COVID‐19 pandemic, a period marked by heightened racial discrimination and mental health challenges for Asian Americans.
- Guided by the Culturally Infused Engagement model, this study examines how individual, familial, ethnic‐cultural, immigrant, and racial stereotype factors together explain mental health service use across Asian American subgroups.
- Depressive symptoms, being female (vs. male), and reduced stigma were associated with higher service use across groups, while language use at home and internalized model minority stereotype had unique impacts on service use across ethnicity, emphasizing the need for tailored mental health interventions for Asian American subgroups.

## INTRODUCTION

During the COVID‐19 pandemic, Asian Americans were disproportionately affected by challenges linked to their racially minoritized status and the discriminatory acts that followed. Prevailing research has shown that Asian Americans were targeted for racially motivated hate crimes and discrimination, not just on the streets but also in the workplace (Cheah et al., 2020; Fang, 2020; Gover et al., 2020; Jeung et al., 2021; Kantamneni, 2020; Kim et al., 2021; Misra et al., 2020; Park et al., 2024; Wu et al., 2021). This unprecedented increase in discrimination experiences due to the COVID‐19 pandemic has deepened existing racial inequities in mental health and access to mental health services (Lin et al., 2023; Xue et al., 2022). Even prior to the pandemic, Asian Americans were least likely to utilize mental health services among all race groups, despite their high rates of mental distress. In 2019, the National Survey on Drug Use and Health (NSDUH) reported that Asian American adults were significantly less likely to receive mental health care compared to other racial groups (e.g., White: 19.8%, Black: 9.8%, Hispanic: 9.7%, Asian: 7%; Substance Abuse and Mental Health Services Administration, 2019); among those with any mental illness, only 23.3% of Asian Americans received mental health services, compared to 50.3% of White, 32.9% of Black, and 33.9% of Hispanic adults. In addition, Jang et al. (2019) found that 28% of Asian American immigrants with serious mental illness reported unmet needs in mental health care. These disparities have worsened during the pandemic. A recent study found that Asian Americans, Hispanic Americans, and Black Americans were significantly less likely than non‐Hispanic Whites to receive any form of mental health treatment or prescription medication and that Asian Americans and Hispanic Americans also had lower outpatient mental health service use than non‐Hispanic Whites during the pandemic's first year (Lin et al., 2023). These inequities in mental health service use pose a significant public health concern.

Furthermore, the diverse socio‐historical and cultural backgrounds of the Asian American community, which encompasses over 50 different ethnicities and speaks more than 100 languages, add layers of complexity to understanding the challenges they face, particularly in terms of mental health service utilization (Jiang et al., 2022). The diversity in language, religion, cultural values, norms, and practices within the group, as well as the rapid population growth of the young Asian Americans in the United States (Budiman, 2021), highlights the growing need for a culturally sensitive approach in identifying the unique barriers and facilitators that influence mental health services utilization, allowing for more effective and culturally tailored interventions to improve mental health outcomes (Huey & Tilley, 2018).

According to the Culturally Infused Engagement (CIE) model (Yasui et al., 2017), these inequities are influenced by factors across various systemic levels, including the micro‐level (such as individual characteristics), the meso‐level (including familial and ethnic‐cultural characteristics), and the macro‐level (encompassing factors related to immigrant status and being part of a racially minoritized group). The CIE model purports that the interplay of these influences at multiple levels shapes the culturally specific patterns of help‐seeking behavior among racially and ethnically diverse individuals. Despite theoretical and empirical studies acknowledging the need to consider multi‐level influences collectively (Park et al., 2023; Yasui et al., 2017), existing research often remains fragmented. That is, studies typically examine individual and contextual factors in isolation, thereby limiting our understanding of their combined impacts on service utilization.

Guided by the CIE model, this study sought to examine multisystem‐level influences on Asian American young adults' mental health service utilization. At the microlevel, we identified several individual‐level factors that are key determinants in mental health service utilization patterns among young Asian Americans. These factors include age, biological sex, socioeconomic status (SES), and mental health symptoms, as highlighted in previous research (Kim & Lee, 2021). While mental health problems in general, irrespective of the types, are known to predict mental health service use among Asian Americans (Kim & Lee, 2021), we specifically focused on general depressive symptoms in the current study due to its high prevalence and severity within both the Filipino American and Korean American communities (Kim et al., 2015). For example, a meta‐analysis of 21,731 Asian American adults found depression rates of 34.4% among Filipino Americans and 33.3% among Korean Americans, notably higher than other Asian American subgroups (Kim et al., 2015). The National Latino and Asian American Study (NLAAS) revealed that older Asian American college students, particularly females or those with severe mental health symptoms, are more likely to utilize specialist services (Ihara et al., 2014). Somewhat inconsistently, another study using the National Health Interview Survey also found that those with higher levels of education and more psychological distress, but younger Asian Americans are more inclined to use mental health services (Balaraman et al., 2023).

Influences at the familial and ethnic‐cultural system levels also significantly shape Asian Americans' beliefs about mental health and mental health service use. Stigma associated with mental health services—prevalent across many cultural and social groups, including Asian Americans—is directly associated with lower mental health service utilization (Kim & Lee, 2021). Additionally, for Asian Americans, adherence to values such as “saving face” (a deep sense of shame and respectability that places significant importance on others' perceptions of oneself and one's family) can deter individuals from seeking mental health services as they strive to maintain their family's reputation and honor (Kim & Lee, 2021; Park et al., 2023). At the macro‐level, the forces of immigration and racism further shape the pathways to help‐seeking for Asian Americans (Pew Research Center, 2012). Immigration‐related factors, such as nativity and English language proficiency, significantly influence mental health service utilization (Kim & Lee, 2021). Specifically, extant research indicates that those born in the United States compared to those born abroad (Balaraman et al., 2023) and with higher English language proficiency (Kim & Lee, 2021) are more likely to use mental health services. This dynamic is further complicated by the macro‐level experiences of Asian American‐specific racial stereotypes, such as the model minority stereotype, which portrays Asian Americans as uniformly successful and problem‐free (Kim & Lee, 2014), and the perpetual foreigner stereotype, which assumes that they are perpetual outsiders in American society (Park et al., 2021). These dual stereotypes act as structural barriers by perpetuating systemic misconceptions about Asian American health needs (Kim, 1999; Kim et al., 2021). For example, societal portrayals of Asian Americans as inherently self‐sufficient obscure the diverse health and socioeconomic challenges within this population. Simultaneously, this depiction may discourage Asian American individuals from seeking help, fearing that doing so might contradict the societal expectations of self‐reliance and resilience (Kim et al., 2021). Such structural biases prolong inequities by framing service use as a failure to meet societal expectations rather than acknowledging systemic obstacles to access (Kim et al., 2021). Moreover, while existing theoretical research (Kim, 1999) underscores the necessity to examine these contrasting stereotypes together due to their interactive influences on the racial experiences of Asian Americans, most existing studies have predominantly focused on the implications of the model minority myth (Kim & Lee, 2014). Yet, the relationship between the perpetual foreigner stereotype and mental health service use, while not directly explored, is pertinent. That is, a study found that awareness of this stereotype has been linked to mental health distress (Park et al., 2021), which could potentially facilitate the use of mental health services.

Although there has been significant progress in understanding how various factors affect mental health service use among Asian Americans, current literature reveals a gap: there is a lack of studies that integrate individual, familial and ethnic‐cultural, immigrant, and racial stereotype factors, with a specific focus on disaggregation by ethnicity. Since these multilevel influences are bounded to the culture and country of origin, it is necessary to approach these factors disaggregated by ethnicity. In this study, we specifically selected Filipino Americans and Korean Americans as our study samples. Both groups report substantial mental health challenges (Kim et al., 2015) but differ notably in their familial, ethnic‐cultural, immigrant, and racial stereotyping experiences in the United States For instance, Filipino American families tend to uphold more traditional values and norms compared to Korean American families, despite being one of the most acculturated ethnic subgroups within Asian American communities when measured by linguistic, occupational, and residential assimilation (Vigdor, 2008). Conversely, Korean Americans represent one of the most socially separated Asian subgroups in the United States (Min, 2006). Regarding racial experiences, Korean Americans, as part of East Asians, are more likely than Filipino Americans to encounter the model minority and perpetual foreigner stereotypes (Park et al., 2021). Filipino Americans who are Southeast Asian Americans with a different phenotype and a relatively darker skin are frequently perceived as Latino/e/x and experience discrimination by other Asians as well (Ocampo, 2016). Despite these differences in ethnic‐cultural, immigrant, and racial stereotyping experiences, few studies have investigated how these intersecting factors uniquely influence mental health service‐seeking behaviors among Filipino Americans and Korean Americans. Moreover, most of the studies on the correlates of mental health service use of Asian Americans are either based on the NLAAS data, which sampled the adult population and was collected before 2010 (Chang et al., 2013; Ihara et al., 2014; Nguyen & Bornheimer, 2014) or are based on small‐size community samples, which restricts the ability to conduct meaningful analyses at the level of specific ethnic or age subgroups. As such, there is a need for more contemporary research that reflects the multi‐layered individual and contextual factors considering the changing demographics of the Asian Americans (Budiman, 2021) and the changing sociopolitical climate, especially in the era of racial awakening (Chang et al., 2023) during the COVID‐19 pandemic.

**Figure 1:**
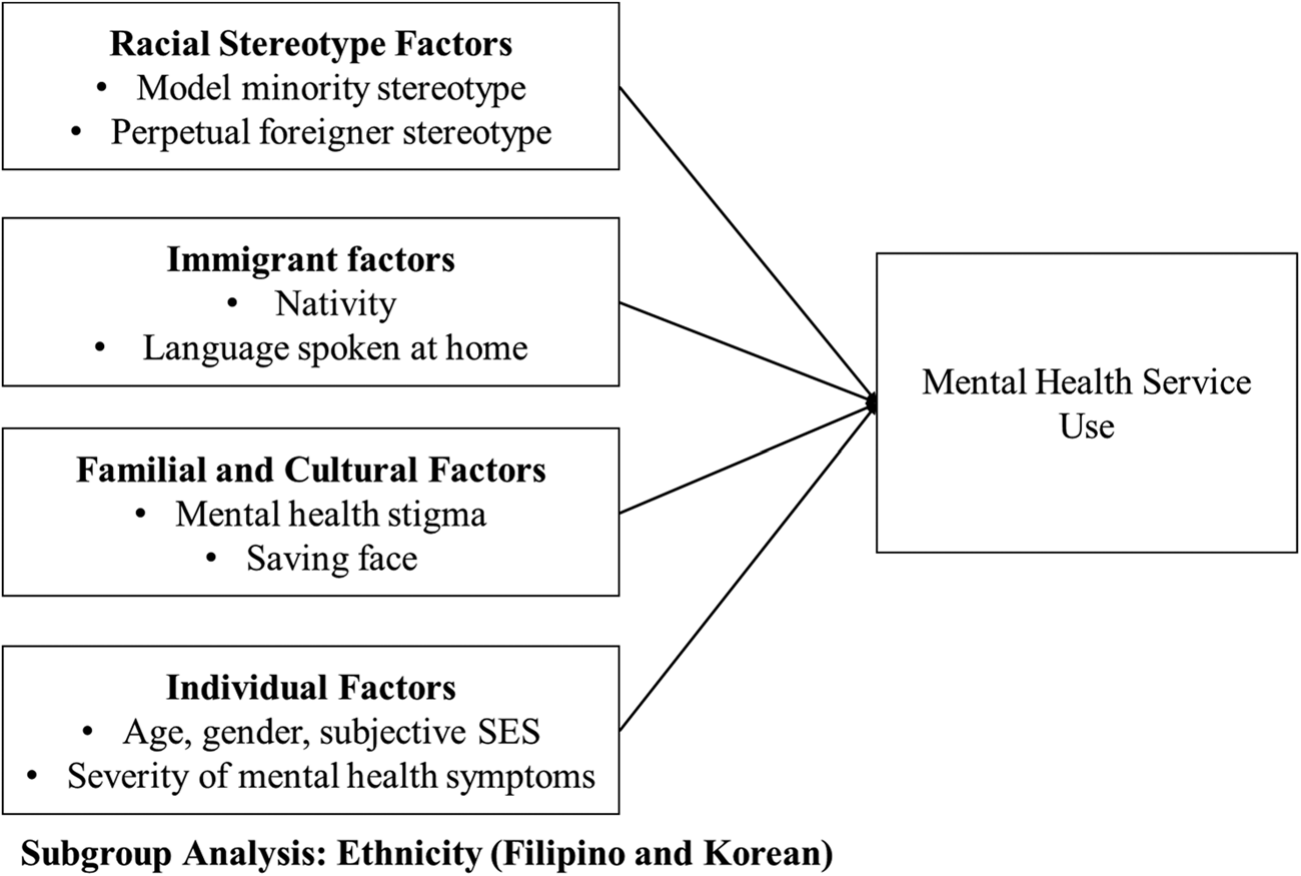
Conceptual model.

To fill this gap, the present study first documented the rates of mental health service use among Filipino American and Korean American young adults during the COVID‐19 pandemic. Secondly, this study examined the roles of individual, familial and ethnic‐cultural, immigrant, and racial stereotype factors on mental health service use across Filipino American and Korean Americans (see Figure 1), thereby shedding light on the diversity within Asian American communities, especially during the unprecedentedly challenging time for Asian American young adults. Based on the CIE model and prior empirical findings discussed earlier, we hypothesized that all individual, familial, ethnic‐cultural, immigrant, and racial stereotype factors included in this study would be significantly associated with mental health service use for both Filipino American and Korean Americans. Specifically, at the individual level, we expected that older individuals, females, and those with higher SES and more severe depressive symptoms would be more likely to use mental health services. At the familial and ethnic‐cultural level, we hypothesized that stigma associated with mental health service use and concerns about saving face related to family matters would be associated with lower mental health service use. At the macro level, we anticipated that individuals more acculturated into American society (e.g., US‐born individuals and those who primarily speak English at home) would be more likely to use mental health services. In addition, we hypothesized that internalizing the model minority stereotype would be associated with less mental health service use, while experiencing the perpetual foreigner stereotype would be related to more mental health service use. Regarding ethnic group differences, we expected no variation in the effects of individual‐level factors on mental health service use. However, as discussed earlier, we anticipated that ethnic‐cultural factors would be more prominent for Filipino Americans. In contrast, immigrant and racial stereotype factors were expected to have a greater impact on Korean Americans than Filipino Americans, as Korean Americans are one of the least acculturated compared to other Asian American subgroups and frequently stereotyped as the model minority and perpetual foreigner as part of the East Asian community.

## METHODS

### Data and sample

This study utilized data from the fourth wave of the Midwest Longitudinal Study of Asian American Families (MLSAAF), collected between April 2021 and April 2022. At the baseline, all participants resided in Chicago and surrounding Midwest areas and were recruited from various sampling sources, including phonebooks, schools, ethnic religious organizations, ethnic grocery stores, and ethnic community organizations. The sampling unit was the family. Only families with an adolescent child, aged between 12 and 17, whose mother self‐identified as Filipino or Korean were eligible to participate. Parents and youth were separately surveyed to protect privacy. At Wave 1, parent participants received an incentive of $40, while child participants received $20. One adolescent child and a primary parent from a family could participate and the participating parents were predominantly biological mothers (92.02% of Filipino Americans and 95.65% of Korean Americans), foreign‐born (90.43% of Filipino Americans and 98.55% Korean Americans), and average age was 45.74 (45.3 for Filipino Americans and 46.21 for Korean Americans). At Wave 4, only youth were followed for the survey. Participants had an option to complete self‐administered surveys either online or in paper format, available in both English and Korean. The majority chose the online survey in English, with only one individual selecting the paper format in English and five opting for the online version in Korean. As an incentive, participants received $50 for completing the online survey and $40 for the paper version. A total of 612 individuals completed the survey in this wave, including 265 Filipino Americans and 347 Korean Americans. These participants, now in their young adult phase, were on average 21 years old, with ages ranging from 18 to 22 years. For additional demographic details of the study participants, please refer to Table 1. The study received approval from the University's Institutional Review Board (IRB).

**Table 1:** Descriptive statistics.

	Foreign‐born (*n* = 214)		US‐born (*n* = 398)		Diff. by nativity		Total (*n* = 612)	
Variables	FA Foreign‐born (FF)	KA Foreign‐born (KF)	FA US‐born (FU)	KA US‐born (KU)	Within FA (FF vs. FU)	Within KA (KF vs. KU)	All FAs	All KAs
**Demographic characteristics**								
Sample size	76 (35.51%)	138 (64.49%)	189 (47.49%)	209 (52.51%)	n.a.	n.a.	265 (43.3%)	347 (56.7%)
Gender (woman)	46 (60.50%)	72 (52.20%)	112 (59.30%)	101 (48.30%)	n.a.	n.a.	158 (59.6%)	173 (49.9%)
Age	21.5 (1.8)	21.8 (1.9)	21.5 (1.8)***	20.9 (1.8)		***	21.5 (1.83)*	21.2 (1.85)
SES	2.8 (0.7)	2.7 (0.8)	3.1 (0.8)***	2.8 (0.9)	**		3.0 (0.7)***	2.76 (0.8)
Depressive symptoms	2.3 (1.0)	2.2 (0.9)	2.2 (0.9)	2.4 (1.0)		+	2.6 (0.93)	2.9 (0.96)
**Predictors**								
Stigma on Mental Health Service Use	2.1 (1.0)	2.0 (0.9)	2.0 (0.9)	2.1 (0.9)			2.0 (0.95)	2.0 (0.85)
Saving Face	3.5 (0.7)***	2.9 (0.5)	3.4 (0.7)***	2.8 (0.6)			3.4 (0.7)*	2.9 (0.6)
Language at home (English)	22 (28.9%)	16 (11.59%)	132 (69.8%)	74 (35.4%)	n.a.	n.a.	154 (58.78%)	90 (26.09%)
Language at home (Ethnic)	29 (38.2%)	108 (78.2%)	23 (12.2%)	87 (41.6%)	n.a.	n.a.	52 (19.85%)	195 (56.52%)
Language at home (Equal)	23 (30.2%)	12 (8.7%)	33 (17.4%)	48 (22.9%)	n.a.	n.a.	56 (21.37%)	60 (17.39%)
MMS–Achievement	3.4 (0.7)	3.2 (0.8)	3.2 (0.8)	3.3 (0.7)			3.3 (0.8)	3.2 (0.8)
MMS–Mobility	2.8 (0.7)***	2.5 (0.8)	2.7 (0.8)***	2.4 (0.7)			2.7 (0.8)***	2.4 (0.7)
PFS	2.6 (0.8)	2.8 (0.9)*	2.1 (0.9)	2.4 (0.8)***	***	***	2.2 (0.9)	2.6 (0.9)***
**Outcome**								
Mental Health Service Use	13 (17.1%)	12(8.69%)	31 (16.40%)	34 (16.27%)	n.a.	n.a.	44 (16.67%)	46 (13.33%)

*Note*: Mean (SD) for continuous variables or sample number (percentage) for categorical variables.

Abbreviations: FA, Filipino Americans; KA, Korean Americans; MMS, Model Minority Stereotype; PFS, Perpetual Foreigner Stereotype.

*** *p* < .001

** *p* < .01

* *p* < .05.

+ *p* .10 indicate *t*‐test statistical differences of the study variables between KF and FF, KU and FU, nativity difference within each ethnicity.

### Measures

Response options for all measures were on an ordinal Likert scale ranging from 1 (e.g., *almost never*) to 5 (e.g., *almost always*), where a higher score indicates a greater level of the construct. All measures, except for the parental report of saving face, were reported by the youth participants. Additionally, biological sex and parental report of saving face were from Wave 1, while all other variables were from Wave 4.

#### Individual‐level factors

The individual‐level factors include age (continuous), biological sex (0 for males, 1 for females), the youth's perceived family SES (rated from 1 for lower class to 5 for upper class), and depressive symptoms. In the MLSAAF survey, depressive symptoms over the past 2 weeks were assessed using 14 items, which included 13 items from the Children's Depressive Inventory (Angold et al., 1995) and 1 item (“I feel like crying a lot of the time”) from the Seattle Personality Questionnaire for Children (Kusche et al., 1988) (*α* = .95 for Filipino Americans; *α* = .95 for Korean Americans). Items were responded using a 5‐point scale from 1 (*almost never*) to 5 (*almost always*).

#### Stigma associated with mental health service use

We measured stigma towards using mental health services with three items from the Indifference to Stigma subscale (Mackenzie et al., 2004). These items reflect discomfort in seeking help from mental health professionals due to concerns about others' opinions and the belief that using mental health services brings shame and stigma (*α* = .77 for Filipino Americans; *α* = .73 for Korean Americans). Items were scored on a 5‐point scale, from 1 (*strongly disagree*) to 5 (*strongly agree*).

#### Parental report of saving face

We assessed the concept of “saving face” using seven items from the MLSAAF (Park et al., 2023). This measure was administered only to parents at Wave 1; therefore, we have included the parental report of “saving face” from Wave 1. Items included statements such as “The reputation of our family is significant” and “When I experience a personal failure, it reflects poorly on the entire family” (*α* = .79 for Filipino Americans; *α* = .80 for Korean Americans). Items were scored on a 5‐point scale, from (*strongly disagre*e) to 5 (*strongly agree*). Each participant's depressive symptoms score was calculated by averaging the scores across 14 items, with higher scores indicating greater levels of depressive symptoms.

#### Nativity

Participants were categorized as foreign‐born (0) or US‐born (1).

#### Language spoken at home

Language spoken at home was assessed with a single item, adapted for each subgroup at Wave 1, asking if participants speak (1) mostly English, (2) more English than Korean/Filipino language, (3) English and Korean/Filipino language equally, (4) more Korean/Filipino Language than English, (5) or mostly Korean/Filipino Language at home. This measure was re‐categorized into three groups: (1) and (2) were combined as “primarily English at home”; (3) was labeled as “both languages equally at home”; and (4) and (5) were grouped as “primarily ethnic language at home” and was then dummy coded with “primarily ethnic language at home” set as the reference group.

#### Model minority stereotype

The Internalization of the Model Minority Myth Measure (Yoo et al., 2010), a 15‐item scale, was used to assess participants' internalization of this stereotype, which includes achievement and mobility aspects. Responses were given on a Likert scale from 1 (*strongly disagree*) to 5 (*strongly agree*). Examples from the achievement aspect include “Asian Americans tend to excel in standardized tests (e.g., SATs) because they prioritize academic achievement.” Mobility aspect items include “Asian Americans face fewer workplace barriers” (Achievement *α* = .93 for Filipino Americans and 0.91 for Korean Americans; Mobility *α* = .82 for Filipino Americans and 0.78 for Korean Americans).

#### Perpetual foreigner stereotype

This stereotype was assessed using the 13‐item Awareness of the Perpetual Foreigner Stereotype scale (Huynh et al., 2011), measuring participants' awareness on a five‐point Likert scale from 1 (*not at all*) to 5 (*very much*). Items included “People don't see me as a typical American” (*α* = .94 for both Filipino Americans and Korean Americans).

#### Past year mental health service use

Youth participants were asked whether they had received any type of treatment for mental health problems in the past year. Response options were No (0) or Yes (1).

### Analytic strategy

We utilized STATA version 17.0 for all data analyses. To begin, we generated descriptive statistics by ethnic groups and conducted analyses to identify any statistical differences among these groups. Additionally, we produced bivariate correlations for each subgroup. To examine both unique and joint contributions of each cluster of predictors of mental health service utilization, hierarchical logistic regression was estimated (Cohen et al., 2003; Hayes, 2017). Specifically, the first step incorporated individual‐level factors, including three demographic variables and depression symptoms. Each subsequent step examined a different cluster of predictors: familial and ethnic‐cultural factors in Step 2, immigrant factors in Step 3, and racial stereotype factors in Step 4, with each step also including the individual factors from Step 1. Finally, all factors were collectively analyzed in Step 5 to examine the robustness of the result from each step. Likelihood Ratio (LR) tests were then conducted to compare each model to Step 1, identifying the model that most efficiently predicts mental health service use (Lewis et al., 2011). We also reported pseudo‐R‐squared values, which, while not directly comparable to R‐squared, provide an indication of overall model fit. Additionally, continuous variables were standardized before analysis by subtracting each variable's mean and dividing by its standard deviation to report the standardized odds ratio (Menard, 2011). The dataset exhibited less than 5% missing data across all variables. To enhance the efficiency of our results, we implemented multiple imputation by chained equations (MICE) on our final analytic sample (265 participants from the Filipino American group and 347 from the Korean American group) and produced 20 imputed datasets.

## RESULTS

Tables 1 and 2 summarize the descriptive statistics among the study variables. Notably, at Wave 4, the rates of past year mental health service use were 16.67% for Filipino American young adults and 13.33% for Korean American young adults. Within each ethnic group, foreign‐born individuals demonstrated a statistically significantly higher awareness of the perpetual foreigner stereotype compared to their US‐born counterparts. Compared to Korean Americans, Filipino Americans exhibited higher rates of parental reports of saving face and a higher internalization of the model minority stereotype, particularly concerning unrestricted mobility. Conversely, Korean Americans reported higher awareness of the perpetual foreigner stereotype than Filipino Americans.

**Table 2:** Correlation between study variables.

Variables	(1)	(2)	(3)	(4)	(5)	(6)	(7)	(8)	(9)	(10)	(11)	(12)	(13)
(1) Age	‐	0.02	−0.06	0.03	−0.08	−0.02	−0.23*	−0.05	−0.08	−0.03	0.05	0.13*	0.13*
(2) Female	0.10	‐	−0.01	0.17*	−0.08	−0.12*	−0.04	0.00	0.08	−0.07	−0.09	0.24*	0.17*
(3) SES	0.07	0.10	‐	−0.07	−0.10	0.05	0.04	0.11*	−0.05	0.04	0.09	−0.03	0.00
(4) Depressive Symptom	−0.11	0.18*	−0.02	‐	0.29*	0.02	0.10	0.01	0.14	−0.04	−0.11*	0.27*	0.32
(5) Stigma	−0.09	−0.02	−0.01	0.27	‐	0.04	0.05	−0.10	0.00	0.12	0.03	0.10	−0.08
(6) Saving Face	−0.05	0.10	−0.04	0.06	0.06	‐	−0.10	0.01	−0.09	0.1	−0.02	−0.06	−0.09
(7) Nativity	0.00	−0.01	0.18*	−0.04	−0.04	−0.06	‐	0.26*	0.18*	0.02	−0.07	−0.22	0.10
(8) Language (English)	0.00	−0.18*	0.16*	−0.09	0.03	−0.07	0.37*	‐	−0.27	−0.01	0.06	−0.10	0.05
(9) Language (Equal)	0.05	0.22*	−0.07	−0.02	−0.13*	0.14*	−0.15*	−0.62	‐	0.00	−0.05	0.03	0.09
(10) MMS–Achievement	−0.04	−0.04	−0.04	−0.05	0.20*	0.12	−0.08	−0.01	−0.05	‐	0.21*	−0.03	−0.17*
(11) MMS–Mobility	0.08	−0.12	−0.08	−0.07	−0.02	−0.01	−0.05	−0.04	0.08	0.28*	‐	−0.14*	−0.07
(12) PFS	0.09	0.22*	−0.12	0.34*	0.17*	0.11	−0.24*	−0.17	0.08	−0.02	−0.11	‐	0.13*
(13) Mental Service Use	0.07	0.22*	0.06	0.17*	−0.11	0.03	0.08	0.13	−0.01	0.00	0.02	0.07	‐

*Note*: Below the diagonal are correlations for Filipino Americans and above the diagonal are correlations for Korean Americans. MMS = Model Minority Stereotype; PFS = Perpetual Foreigner Stereotype.

* *p* <.05

Table 3 presents the results of the logistic regression analysis, which were structured according to the following analysis steps: individual (Step 1), familial and ethnic‐cultural (Step 2), immigrant (Step 3), racial stereotype (Step 4), and all (Step 5) factors. Regarding the LR test results, only the inclusion of variables in Step 2 significantly improved the model fit for Filipino Americans. For Korean Americans, model fit showed significant improvement in Steps 2, 4, and 5 compared to Step 1. Specifically, in the initial step focusing on individual‐level factors (Step 1; *Pseudo R*² = 0.089 for Filipino Americans and 0.114 for Korean Americans), females exhibited higher rates of mental health service use than males across both ethnic groups. Additionally, depressive symptoms were significantly linked to more mental health service use in both groups. Age showed a significant association with more mental health service use exclusively among Korean Americans. For both ethnic groups, the addition of the familial and ethnic‐cultural factors (Step 2; *Pseudo R*² = 0.135 for Filipino Americans and 0.163 for Korean Americans) provided the greatest improvement to the model fit compared to the inclusion of other factors. Specifically, greater stigma toward mental health service use was significantly associated with lower service use for both ethnic groups. The inclusion of the immigrant factors (Step 3; *Pseudo R²* = 0.093 for Filipino Americans and 0.133 for Korean Americans) provided less model fit improvement for mental health service usage than the familial and ethnic‐cultural factors, thought it contributed more than the racial stereotype factors among Filipino Americans. Conversely, among Korean Americans, the immigrant factors provided the least model fit improvement. Specifically, Filipino Americans who primarily used English at home (vs. ethnic language at home) were more likely to utilize mental health services. In contrast, the addition of the racial stereotype factors (Step 4; *Pseudo R*² = *0.092* for Filipino Americans and 0.143 for Korean Americans) provided the least model fit improvement among Filipino Americans but represented the second most explanatory factor among Korean Americans. Specifically, adherence to the achievement aspect of the model minority stereotype was linked to less use of mental health services among Korean Americans. All predictor clusters were incorporated in the final model (Step 5; *Pseudo R*² = 0.142 for Filipino Americans and 0.197 for Korean Americans). All significant predictors identified in the earlier steps remained significant in this comprehensive model, highlighting their robust association with mental health service utilization across the study groups.

**Table 3:** Hierarchical logistic regressions (standardized odds ratio (95% [CI]).

	Filipino Americans					Korean Americans				
	Step1	Step2	Step3	Step4	Step5	Step1	Step2	Step3	Step4	Step5
*Individual factor*										
Age	1.17	1.16	1.16	1.17	1.14	1.39*	1.36*	1.50**	1.39*	1.44*
	(0.88–1.56)	(0.87−1.55)	(0.87−1.56)	(0.88−1.56)	(0.84−1.54)	(1.07−1.82)	(1.03−1.78)	(1.14−1.99)	(1.06−1.83)	(1.08−1.93)
Woman (Ref. Man)	2.54**	2.45**	2.92**	2.66**	2.94**	1.94*	1.65	1.99*	1.89*	1.65^+^
	(1.39−4.65)	(1.33−4.52)	(1.54−5.54)	(1.43−4.94)	(1.52−5.68)	(1.14−3.31)	(0.96−2.86)	(1.16−3.42)	(1.09−3.28)	(0.93−2.91)
SES	1.09	1.10	1.00	1.10	1.03	1.09	1.07	1.07	1.12	1.08
	(0.83−1.44)	(0.83−1.45)	(0.75−1.34)	(0.83−1.46)	(0.76−1.38)	(0.84−1.42)	(0.82−1.41)	(0.82−1.40)	(0.85−1.47)	(0.82−1.42)
Depressive Symptoms	1.39*	1.53**	1.46	1.42*	1.62	2.02***	2.36***	1.95***	2.02***	2.22***
	(1.06−1.83)	(1.14−2.04)	(1.10−1.94)	(1.06−1.91)	(1.18−2.25)	(1.55−2.62)	(1.76−3.16)	(1.49−2.54)	(1.54−2.66)	(1.63−3.03)
*Familial and Cultural*										
Stigma		0.72*			0.68*		0.67			0.70*
		(0.54−0.97)			(0.50−0.94)		(0.50−0.89)			(0.51−0.94)
Saving Face		1.04			1.04		0.80			0.84
		(0.78−1.40)			(0.76−1.42)		(0.61−1.04)			(0.64−1.10)
*Immigrant factor*										
Nativity			1.13		1.16			1.65		1.80
			(0.57−2.25)		(0.57−2.38)			(0.90−3.02)		(0.95−3.41)
Language at home (Eng.)			2.89*		2.83*			1.30		1.19
			(1.21−6.90)		(1.17−6.84)			(0.67−2.51)		(0.60−2.37)
Language at home (Equal)			1.60		1.38			1.33		1.19
			(0.61−4.22)		(0.51−3.75)			(0.64−2.76)		(0.56−2.52)
*Racial Stereotype factor*										
MMS–Achievement				1.02	1.12				0.68**	0.70*
				(0.77−1.35)	(0.83−1.52)				(0.52−0.89)	(0.53−0.93)
MMS–Mobility				1.11	1.11				0.98	1.02
				(0.83−1.48)	(0.83−1.50)				(0.74−1.28)	(0.77−1.35)
PFS				0.95	1.05				1.02	1.12
				(0.70−1.28)	(0.76−1.44)				(0.77−1.35)	(0.83−1.51)
*Pseudo R2*	0.089	0.135	0.093	0.092	0.142	0.114	0.163	0.133	0.143	0.197
*Log Likelihood*	−108.41	−102.83*	−107.88	−108.00	−102.09	−120.05	−113.42*	−117.43	−116.05*	−108.74*

*Note*: Log Likelihood ratio test was conducted between step 1 and other models, respectively.

Abbreviations: MMS, Model Minority Stereotype; PFS, Perpetual Foreigner Stereotype.

+ *p* < .1

* *p* < .05

** *p* < .01

*** *p* < .001.

### Sensitivity analysis

Given that data collection occurred during the COVID‐19 pandemic, we examined the consistency of study findings after accounting for COVID‐19‐specific discrimination experiences. To measure COVID‐19‐related racial discrimination, we included nine items: seven from the Pandemic Asian Discrimination Scale (Liu et al., 2020) and two additional items by MLSAAF to capture vicarious COVID‐19 racial discrimination experiences (e.g., “Someone close to me has been verbally assaulted because they are Asian due to COVID‐19” and “Someone close to me has been physically assaulted because they are Asian due to COVID‐19”). Each item was rated on a dichotomous scale, with 0 indicating no occurrence and 1 indicating occurrence of these experiences, and an aggregate score (ranging from 0 to 9) represented the overall level of discrimination experiences. The mean scores for COVID‐19‐related racial discrimination were 6.48 (SD = 2.25) for Filipino Americans and 6.83 (SD = 2.04) for Korean Americans. Next, to evaluate the sensitivity of the study findings to the inclusion of years living in the United States, we used immigration generation status as a proxy, rather than adding years living in the United States directly, due to the high correlations among nativity, age, and years living in the United States. The study participants were categorized into three generational groups: 1st generation (foreign‐born, arrived in the United States after age 12), 1.5 generation (foreign‐born, arrived in the United States at age 12 or younger), or 2nd generation (US‐born). Among the foreign‐born participants, 32 were 1st generation (12 Filipino Americans and 20 Korean Americans), and 182 were 1.5 generation (64 Filipino Americans and 118 Korean Americans) (Generation 1.5 students: Recognizing an overlooked population, n.d). Additionally, we evaluated whether including English proficiency, measured by the mean of two items (e.g., “How well do you understand English?” rated from 1 = *Not at all* to 5 = *Very well*), affected the study findings. The results indicated that the inclusion of COVID‐19‐related discrimination, immigration generation, and English proficiency did not significantly alter the study outcomes (see Table 4, S1).

**Table 4:** Sensitivity analysis [standardized odds ratio (95% CI)].

	Filipino Americans		Korean Americans	
	S1	S2	S1	S2
*Individual Factor*				
Age	1.08	1.06	1.24**	1.23**
	(0.91−1.27)	(0.90−1.25)	(1.05−1.45)	(1.05−1.44)
Woman (Ref. Man)	2.95**	3.22***	1.62	1.62
	(1.49−5.81)	(1.63−6.34)	(0.91−2.88)	(0.90−2.90)
SES	1.03	1.03	1.10	1.08
	(0.68−1.55)	(0.68−1.57)	(0.78−1.55)	(0.77−1.52)
Depressive Symptoms	1.64**	1.63**	2.31***	2.28***
	(1.14−2.36)	(1.13−2.36)	(1.66−3.21)	(1.64−3.16)
*Familial and Cultural*				
Stigma	0.67*	0.64*	0.68*	0.66*
	(0.47−0.94)	(0.45−0.90)	(0.47−0.98)	(0.46−0.95)
Saving Face	1.10	1.15	0.74	0.76
	(0.71−1.70)	(0.74−1.78)	(0.45−1.20)	(0.46−1.24)
*Immigrant factors*				
1.5 gen (ref. 1st gen)	0.71		0.67	
	(0.12−4.17)		(0.16−2.83)	
2nd gen (ref. 1st gen)	0.79		0.57	
	(0.35−1.77)		(0.29−1.11)	
Language at home (Eng.)	3.12*	1.00	1.19	2.47
	(1.22−7.98)	(0.23−4.46)	(0.60−2.39)	(0.68−9.05)
Language at home (Equal)	1.55	0.52	1.22	0.69
	(0.54−4.42)	(0.12−2.38)	(0.57−2.60)	(0.13−3.71)
English Proficiency	1.24		1.73	
	(0.32−4.88)		(0.74−4.02)	
Nativity (ref. Foreign born)		0.22		2.08
		(0.04−1.31)		(0.92−4.67)
Nativity × Language at home (English)		9.90*		0.40
		(1.13−86.88)		(0.09−1.83)
Nativity × Language at home (Equal)		9.92*		2.00
		(1.04−94.73)		(0.30−13.31)
*Racial Stereotype factors*				
MMS–Achievement	1.18	1.16	0.62*	0.70*
	(0.80−1.74)	(0.78−1.71)	(0.43−0.90)	(0.53−0.93)
MMS–Mobility	1.10	1.08	1.02	1.02
	(0.73−1.65)	(0.72−1.63)	(0.68−1.54)	(0.77−1.35)
PFS	1.01	1.05	1.19	1.12
	(0.68−1.50)	(0.70−1.59)	(0.83−1.72)	(0.83−1.51)
Covid‐ 19 Specific discrimination	0.98		1.01	
	(0.86−1.11)		(0.89−1.14)	

Abbreviations: MMS, Model Minority Stereotype; PFS, Perpetual Foreigner Stereotype; S1, Sensitivity analysis adjusting for immigration status and COVID‐19 specific discrimination; S2, Sensitivity analysis by nativity status.

*** *p* < .001

** *p* < .01

* *p* < .05.

**Figure 2:**
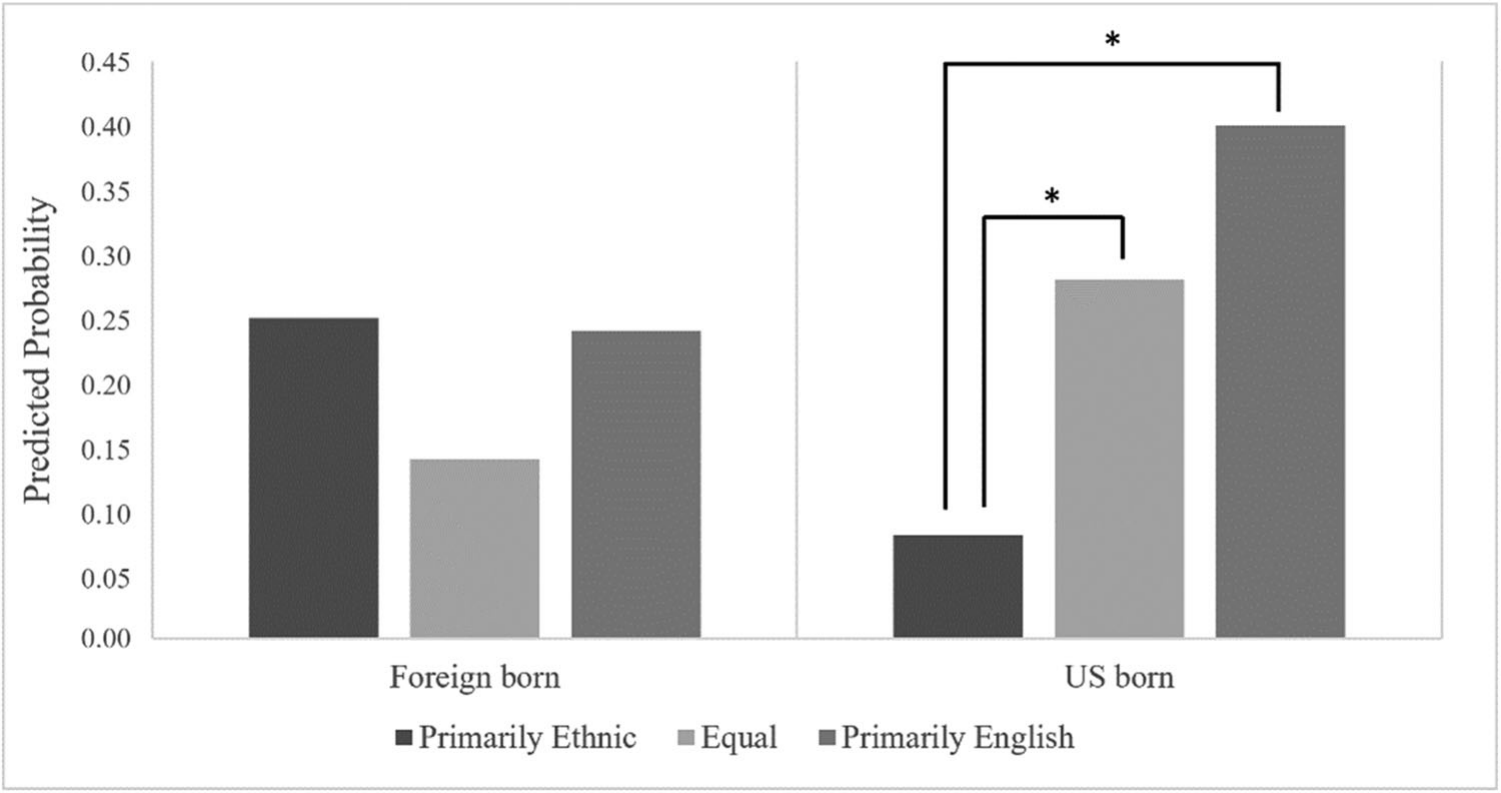
Mental health service use among Filipino Americans by nativity. *p < .05.

In addition, to investigate whether the study results vary by nativity status, interaction terms (each predictor × nativity) were added to the Step 5 model, respectively. The impact of most factors on the utilization of mental health services did not significantly vary by nativity status. However, the influence of the language spoken at home among Filipino Americans differed based on whether they were US‐born or foreign‐born. Specifically, among US‐born individuals, those who primarily speak English at home, as well as those who use both English and an ethnic language equally, were significantly more likely to utilize mental health services compared to those who primarily use an ethnic language at home (Nativity × Language at home (English) *OR* = 9.90, *p* < .05; Nativity × Language at home (Equal) *OR* = 9.92, *p *< .05). Conversely, for the foreign‐born population, the choice of language spoken at home did not have a significant association with the use of mental health services (see Table 4, S2 and Figure 2).

## DISCUSSION

This study aimed to document mental health service use patterns in the context of unmet mental health needs—the lowest service utilization despite high mental distress among Filipino American and Korean American young adults during the COVID‐19 pandemic. Specifically, we examined the collective effects of multi‐dimensional influences on their mental health service use. Our analysis revealed no discernible difference in mental health service utilization rates between Filipino Americans and Korean Americans based on the current MLSAAF data. However, the observed rates for Filipino Americans (16.67%) and Korean Americans (13.33%) in the 2022 MLSAAF data were significantly higher than the pre‐pandemic rates of 7% reported in 2019 NSDUH data for Asian American young adults (18 or older) (Substance Abuse and Mental Health Services Administration, 2019). Moreover, the mental health service use rates from the current study conducted in 2022 indicate that our sample reflects typical characteristics of Asian Americans, aligning with the rates of Asian Americans age 18 or older reported in the nationally representative 2022 data from NSDUH (12.3%) (Substance Abuse and Mental Health Services Administration, 2023). This suggests a notable shift in the patterns of mental health service engagement among these groups during the pandemic.

Furthermore, the current study underscored both the similarities and differences in the factors related to mental health service utilization among Filipino American and Korean American young adults, emphasizing the importance of customized interventions to meet the specific mental health care needs of various Asian American subgroups. Similarities across Filipino American and Korean American young adults included individual mental health symptoms as a significant predictor of mental health service utilization, even after considering all factors in the model, which is in line with existing literature (Balaraman et al., 2023; Ihara et al., 2014; Kim & Lee, 2021). Interestingly, as shown in the bivariate correlation in Table 2, we found that for both ethnic groups, the same mental health symptoms were significantly correlated with higher stigma associated with mental health service use, contrasting with findings in existing literature (Kim & Lee, 2021). Given that these findings are based on bivariate relationships, the results may change in multiple regression analyses after accounting for other key covariates. Future research should further investigate this relationship by using multivariate models that adjust for other important covariates to better understand the link between mental health symptoms and stigma associated with mental health service use.

Differences emerged between Filipino American and Korean American young adults, where Filipino females were significantly more likely to use mental health services. However, for Korean Americans, biological sex was not a significant predictor in the comprehensive model, although it was significant when considering only individual‐level factors, which is consistent with prior research (Ihara et al., 2014; Kim & Lee, 2021). This finding highlights the importance of acknowledging gender‐specific experiences within ethnic groups in mental health service use. It is possible that males may underutilize mental health services due to cultural stereotypes surrounding masculinity and a reluctance to express vulnerability (Wong et al., 2017; Yousaf et al., 2015). Future research should conduct a deeper exploration into the gendered dynamics of mental health service‐seeking behavior.

In examining familial and ethnic‐cultural factors, our findings revealed that stigma associated with mental health service use was a significant predictor of service use across Filipino Americans and Korean Americans, aligning with existing research (Kim & Lee, 2021). Contrary to expectations, however, parental reports of saving face did not predict service use for either group. It is possible that while Filipino American and Korean American young adults may experience pressure from their parents to keep family matters private and maintain face, potentially contributing to mental health distress (Chong, 2019; Kong et al., 2020), this pressure may not be strong enough to deter them from seeking help. It is also possible that younger generations of Filipino Americans and Korean Americans are increasingly able to navigate or even challenge saving‐face narratives regarding mental health. This may be consistent with broader Gen‐Z trends, where mental health is discussed more openly and seeking professional help is viewed as a form of self‐empowerment rather than shame (Bizzotto et al., 2023).

Although the immigrant factors as a whole did not contribute substantially to explaining mental health service use patterns, several individual predictors were statistically significant. Specifically, place of birth was not a significant predictor of mental health service use for both groups in our logistic regression models. However, it was marginally significant (*p* < .1) for Korean American young adults in the comprehensive model, and for Filipino American young adults when considered in conjunction with the language spoken at home in the interaction model. Specifically, US‐born Korean American young adults reported marginally higher mental health service usage compared to their foreign‐born counterparts. The MLSAAF data found that US‐born Korean American youth reported more depressive symptoms (Park et al., 2021) and their higher use of MH services during young adulthood may reflect a response to these increased depressive symptoms. Additionally, a higher level of acculturation into American society and familiarity with the US healthcare system among the US‐born likely contribute to this increased usage of mental health services.

Language spoken at home was significantly related to mental health service use among Filipino American young adults, but not among Korean American young adults. Specifically, within Filipino Americans, this trend was particularly pronounced among the US‐born. That is, the sensitivity analysis revealed that US‐born Filipino American young adults who primarily spoke an ethnic language at home were less likely to utilize mental health services, aligning with findings from existing literature (Kim & Lee, 2021). This observation may be attributed to the high level of acculturation among Filipino Americans, especially those born in the United States; according to current MLSAAF data, 66% of Filipino Americans mainly use English at home (70% of the US‐born vs. 30% of the foreign‐born), in contrast to 46% of Korean Americans (35% of the US‐born vs. 11% of the foreign‐born) who predominantly communicate in their ethnic language. Thus, for Filipino Americans, particularly those who are US‐born and generally show higher acculturation rates compared to Korean Americans and foreign‐born Filipino Americans, speaking an ethnic language at home may suggest a considerably lower level of acculturation into American society. This, in turn, is a crucial factor in their lower likelihood of seeking mental health services. Considering that language match is an important predictor of mental health service use among Asian Americans (Sue, 1998), the limited availability of linguistically concordant mental health services represents a structural barrier that could mediate the relationship between language use and service utilization (Alegría et al., 2023). While the Culturally and Linguistically Appropriate Services standards have been established (Koh et al., 2014), their legal mandate on providing language access is restricted to organizations receiving federal funding (Applebaum & Robbins, 2016). This systemic limitation creates a service gap for individuals who prefer or require communication in their non‐English primary language.

The impact of racialization was reflected in how the internalization of the model minority stereotype affected mental health service utilization among Korean Americans; however, this was not the case for Filipino Americans, aligning with prior research suggesting that these Asian American‐specific stereotyping experiences may be less relevant to Filipino Americans (Kim & Lee, 2014). This variation may stem from differences at the ethnic subgroup level. Specifically, Korean Americans are East Asians, who are commonly associated with the model minority stereotype. In contrast, Filipino Americans, with their frequently Spanish‐sounding last names from three centuries of Spanish colonization and typically darker skin tones, are often not categorized as such and may instead be mistaken for Latinx Americans (Ocampo, 2016). The pressure of meeting high, yet unachievable, expectations from schools and the larger society, solely based on being East Asian Americans, may make the internalization of this stereotype particularly harmful for Korean Americans. This aligns with our previous study on mental health outcomes, which showed that the internalized model minority myth was particularly harmful to Korean American youth in comparison to Filipino American youth (Park et al., 2021).

Our study adds to the existing understanding by demonstrating how a seemingly positive stereotype, such as being hardworking and intelligent, can not only increase psychological distress but also hinder individuals from seeking necessary help. More importantly, the current study identified the model minority stereotype as a substantial structural barrier maintaining inequities in mental health service access. The model minority stereotype impacts multiple levels, including healthcare funding, service provision, and public awareness, contributing to a lack of culturally tailored mental health resources (Kim et al., 2021). Acknowledging the structural role of stereotypes like the model minority stereotype underscores the need for a multi‐level approach to mental health disparities, moving beyond attributing service underutilization solely to individuals or families.

While the perpetual foreigner stereotype did not emerge as a significant predictor in the logistic regression models for both ethnic groups, it was significantly correlated with increased mental health service use among Korean American young adults (see Table 2). Regardless of ethnic background, heightened awareness of this stereotype has been linked to increased mental distress, such as depression and suicidal ideation, in our prior research using multiple regression analyses (Park et al., 2021) and in the current study's bivariate correlations (See Table 2). This suggests that the elevated level of mental distress associated with this stereotype may lead to greater utilization of mental health services. In terms of ethnic group differences, this finding is consistent with our previous study, which indicated a notably higher awareness of the perpetual foreigner stereotype among Korean American young adults compared to Filipino American counterparts (Park et al., 2021). It is possible that the experiences and impacts of the perpetual foreigner stereotype may be more acute among Korean Americans, impacting their mental health and service utilization patterns more significantly than Filipino Americans.

## LIMITATIONS

The limitations of this study should be acknowledged. First, this study used cross‐sectional data within a longitudinal study, with the exceptions of saving face and language spoken at home, which were derived from earlier parental and youth reports, respectively. Consequently, our findings illustrate correlational rather than temporal relationships. Nonetheless, this research contributes significantly to the literature by examining the interplay of various factors—including individual demographic characteristics, family dynamics, cultural influences, immigrant status, and racial stereotypes—and their impact on mental health service utilization. Future research employing longitudinal methodologies is recommended to elucidate more causal relationships within these dynamics. Second, the generalizability of our findings is limited to the regional sample from which our data were drawn. This poses a potential limitation in applying our results to other U.S. regions with differing demographic profiles, economic conditions, and cultural attitudes towards immigrants and Asian Americans. Despite this, the study offers valuable insights into the Midwestern context, a geographical area often underrepresented in research focusing on Asian American populations, which predominantly centers on coastal regions. Third, our study's focus on Filipino Americans and Korean Americans, particularly those in emerging adulthood, does not encapsulate the entire spectrum of Asian American experiences. Considering the rich diversity within Asian American subgroups—each characterized by unique familial and cultural norms, racial stereotyping experiences in the United States, and varied life‐course trajectories—future research endeavors should aim to encompass a broader range of these groups. This inclusive approach is essential to develop a more comprehensive understanding of the factors impacting mental health service‐seeking behaviors across diverse Asian American populations and life stages. Fourth, important structural factors—such as participants' health insurance coverage for mental health care and the availability of culturally and linguistically relevant providers—were not included in this study as such information was not available in the MLSAAF survey. The absence of such measures may limit the interpretation of our findings and potentially obscure the systemic barriers that contribute to disparities in service use among Asian American young adults. Future waves of the MLSAAF should consider incorporating these variables to more accurately capture macro‐level determinants of mental health service engagement. Lastly, adding some clusters of predictors to the step 1 model appeared to contribute minimally to the improvement of the overall model fit. However, we believe that interpreting the effects of each significant predictor in the model, even if the entire cluster is not significant, provides meaningful insights into the limited literature on mental health service use among Asian American subgroups.

## CONCLUSION

Despite its limitations, this study is notable for being among the few that have documented the multiple systemic factors related to mental health service use during the COVID‐19 pandemic among Filipino American and Korean American young adults in the Midwestern US. The unusually high rates of mental health service use during the pandemic underscore the critical need to address mental health service use among Asian Americans. However, it is important to approach this by recognizing both the shared and unique determinants of mental health service use among Filipino American and Korean American young adults. Ethnic specific influences on mental health service utilization, such as the use of ethnic language at home as a barrier for US‐born Filipino Americans, whereas the internalized model minority stereotype as a significant obstacle in seeking help for Korean Americans, highlight the need for developing interventions tailored to the ethnic‐specific experiences in mental health service utilization among Asian American young adults in the increasingly diverse and growing Asian American population. As we face an increasingly diverse US population, it is imperative for research and practice to consider the complex, multisystemic influences on mental health, an endeavor for future research that aims to reduce racial disparities in mental health.

## AUTHOR CONTRIBUTIONS

The first and second authors, as equal contributors, conceived, designed, and conducted the study, including the analysis and interpretation of data, and writing the manuscript. The third author contributed to the conceptual framework of the study by providing feedback and writing the Introduction section. The fourth author provided the dataset and feedback throughout the study. The fifth, sixth, and seventh authors provided feedback on the conceptual framework of the study. All authors read and approved the final manuscript.

## CONFLICT OF INTEREST STATEMENT

The authors declare no conflicts of interest.

## ETHICS STATEMENT

This study was conducted in compliance with ethical standards. All procedures of the study including data collection and analyses were approved by the Institutional Review Board of the third author's university to ensure the proper protection of human subjects, including confidentiality of the data. Informed consent was obtained from all individual participants included in the study.

## Data Availability

The datasets analyzed in the current study are not publicly available but can be available from the fourth author if certain conditions are met.
